# Is early ventricular dysfunction or dilatation associated with lower mortality rate in adult severe sepsis and septic shock? A meta-analysis

**DOI:** 10.1186/cc12741

**Published:** 2013-05-27

**Authors:** Stephen J Huang, Marek Nalos, Anthony S McLean

**Affiliations:** 1 Department of Intensive Care Medicine, Nepean Hospital, Nepean Clinical School, University of Sydney, Penrith NSW 2750, Australia

## Abstract

**Introduction:**

Reversible myocardial depression occurs early in severe sepsis and septic shock. The question of whether or not early ventricular depression or dilatation is associated with lower mortality in these patients remains controversial. Most studies on this topic were small in size and hence lacked statistical power to answer the question. This meta-analysis attempted to answer the question by increasing the sample size via pooling relevant studies together.

**Methods:**

PubMed, Embase (and Medline) databases and conference abstracts were searched to July 2012 for primary studies using well-defined criteria. Two authors independently screened and selected studies. Eligible studies were appraised using defined criteria. Additional information was sought the corresponding authors if necessary. Study results were pooled using random effects models. Standardized mean differences (SMD) between survivor and non-survivor groups were used as the main effect measures.

**Results:**

A total of 62 citations were found. Fourteen studies were included in the analysis. The most apparent differences between the studies were sample sizes and exclusion criteria. All studies, except four pre-1992 studies, adopted the Consensus definition of sepsis. Altogether, there were >700 patients available for analysis of the left ventricle and >400 for the right ventricle. There were no significant differences in left ventricular ejection fractions, right ventricular ejection fractions, and right ventricular dimensions between the survivor and non-survivor groups. When indexed against body surface area or body height, the survivors and non-survivors had similar left ventricular dimensions. However, the survivors had larger non-indexed left ventricular dimensions.

**Conclusion:**

This meta-analysis failed to find any evidence to support the view that the survivors from severe sepsis or septic shock had lower ejection fractions. However, non-indexed left ventricular dimensions were mildly increased in the survivor group but the indexed dimensions were similar between the groups. Both survivors and non-survivors had similar right ventricular dimensions.

## Introduction

The phenomenon of sepsis related myocardial dysfunction has been recognized for over three decades [1]. One of the earliest studies by Weisel et al. in 1977, using pulmonary artery catheter thermodilution method measured left ventricular stroke work index, observed that not only was left ventricular depression common in patients with sepsis, but was also potentially reversible [2]. They also found that the left ventricular systolic function was reduced in non-survivor. Six years later, Hoffman et al., using gated cardiac scintigraphy, documented that right ventricular ejection fraction (RVEF) was depressed in patients with septic shock compared to normal [3]. It was unclear from this study whether the survivors exhibited a better baseline right ventricular function. The most quoted study on this subject is Parker and co-authors' 1984 landmark paper which reported approximately half of the 20 patients with septic shock in the study had severely reduced baseline left ventricular ejection fraction (LVEF), and that the mean LVEF was paradoxically lower in survivors [4]. Using radionuclide cineagiography, they also showed that survivors had dilated left ventricles (LV). In a subsequent study, Parker et al. further found that survivors of septic shock also had depressed initial RVEF, a finding that was not shared by Vincent et al., who noted that survivors from septic shock had markedly higher initial RVEF in two studies [5,6].

While many physicians believe poor LVEF have better outcome in patients with sepsis, some conflicting data have started to emerge in recent years. For example, Vieillard-Baron et al., by examining the hemodynamic parameters in septic shock patients, found that the initial LVEF were similar in survivors and non-survivors [7]. Pulido et al. also failed to find any association between 30- or 100-day mortality with initial ventricular dysfunction [8]. Some studies also casted doubts about the earlier claims that survivors from severe sepsis or septic shock have larger ventricular size [7,9].

Despite all these studies, the debates of whether or not ventricular dysfunction or dilatation (increased ventricular cavity size) is associated with lower mortality in severe sepsis or septic shock continue. One of the main reasons is the small sample size in majority of these studies lacked the statistical power to detect genuine differences in effect sizes. In light of this continuing uncertainty, we conducted this meta-analysis to assist in clarifying the situation. The goals of this meta-analysis are: (1) to critically appraise the best available observational studies that correlated venticular function and/or dimension with mortality in adult patients with severe sepsis or septic shock; (2) to synthesize the relevant data and determine if ventricular dysfunction or dilatation is associated with lower mortality rate; and (3) to list the main difficulties encountered in past studies and to propose some suggestions for future studies on this subject.

## Methods

### Study identification

Our search strategy began by breaking the research question down to: (1) patient type (study population); (2) indicator or indices; and (3) outcomes. For patient type, we included only severe sepsis or septic shock; for indicator, we used myocardial depression and any synonyms; for outcomes, we looked for mortality and any synonyms. Primary studies dated July 2012 and earlier were searched using PubMed and Embase (including Medline). The following terms (some with truncation) were searched as key and text words: 'Sepsis OR septic shock [title]' AND '((Ventricular OR cardiac OR myocardial) AND (failure OR depression OR dysfunction OR function OR impairment OR dimension OR dilat*)) OR (ejection fraction) [title]' AND '(death OR mortality OR surviv* OR outcome OR predict* OR prognos*)'. We limited the search to human studies, English language, clinical study, outcome study, clinical trials, article, article in press, conference abstract, or conference paper. The reference lists of all eligible studies were examined to search for other relevant studies.

### Study eligibility

Two investigators (SJH and MN) independently reviewed the titles and abstracts of all relevant studies; all potential relevant studies were retrieved. When disagreement occurred, the reviewers resolved the discrepancies by consensus. Selection criteria used to identify studies were: prospective or retrospective observational studies; adult patients with either severe sepsis or septic shock; the initial measurements must be obtained objectively and as early as practicable; and used mortality as one of the outcome. The 1992 Consensus definitions of sepsis and organ failure was applied in all studies except for those published before 1992 [10]. For these pre-1992 studies, all of which recruited septic shock patients, we adopted the definitions of septic shock as specified by the primary authors and the minimum criteria had to be: (1) positive blood culture or documented source of infection; (2) hypotension; and (3) clinical evidence of tissue hypoperfusion (such as lactic acidosis, oliguria, or decreased mental state). Studies were excluded if they: included minors (aged <18 years), were unclear about the methods of recruitment, duplicated studies, or contained insufficient information to extract the data.

### Appraisal of study quality

Most of the studies were descriptive in nature and were not designed as true prognosis studies. We therefore modified an appraisal checklist for prognosis study developed by the Centre for Evidence Based Medicine, Oxford, to assess the quality of the studies [11]. Details of the appraisal items are shown in Table 1. Studies that received more than one 'No' or 'Unclear' to the questions were excluded.

**Table 1:** Appraisal checklist for study quality.

*Recruitment* Was the study cohort representative of adult population with severe sepsis or septic shock? (*Yes/No/Unclear*) Were the patients recruited at an early point in the course of sepsis? (*Yes/No/Unclear*) Were eligible patients recruited consecutively? Were all eligible patients within the study period recruited? (*Yes/No/Unclear*)
***Outcome and follow-up*** Was 'mortality' clearly defined? (*Yes/No/Unclear*) Was patient follow-up sufficiently long and complete given the type of mortality defined? (*Yes/No/Unclear*)

***Measurement*** Were all measurements obtained objectively using an established method? (*Yes/No/Unclear*) Were the initial measurements made sufficient early in the course of sepsis? (*i.e*. within 24 hours of diagnosis or onset of symptoms) (*Yes/No/Unclear*) Did the patients receive the same number of measurements using the same method at the same stage? (*Yes/No/Unclear*) Were the assessors blinded from the measurements or outcome? (*Yes/No/Unclear)*

### Data abstraction

Data abstraction was conducted by two investigators in each study to obtain information on year of publication, country, patients' demographics, sample size, ventricular function and dimension data, mortality, and other clinical data. Any disagreements were resolved by consensus. We contacted the primary investigators to provide further information when data were missing or unclear. The studies were excluded if the authors did not respond.

The following data were abstracted: for LV systolic function, LVEF and LV fractional area contraction; for LV dimension, LV end-diastolic diameter (LVEDD) or volume (LVEDV); for RV systolic function, RVEF and RV stroke volume change; and for RV dimension, RV end-diastolic diameter (RVEDD), volume (RVEDV), or area (RVEDA).

### Data analysis

The meta-analysis was performed using STATA with *mais *package. Patients were divided into two groups: survivors and non-survivors from severe sepsis or septic shock. Data from different studies were combined to obtain a pooled outcome effect measure and unless specified otherwise, all data were expressed as standardized mean difference (SMD) (95% confidence interval (CI)), SMD, which is the weighted mean difference (survivors minus non-survivors) of the outcome measure (for example, LVEF). An overall SMD of zero represents no difference between the survivors and non-survivors. A negative value indicates survivors had smaller value, and a positive value indicates survivors had larger value. Since outcome data were presented in form of continuous data, and different studies might use different variable outcomes (for example, LVEDD or LVEDV for ventricular size) or different methods (for example, radionuclide *vs*. echocardiography) to obtain the same variable outcome, the SMD was calculated as described by DerSimonian and Laird's random effects model [12].

Heterogeneity was tested using Cochran's Q test. A *P *value of < 0.05 was considered as indication of significant heterogeneity. I^2 ^statistics were computed from Cochran's Q test as described by Higgins et al. [13]. I^2 ^is the percentage variation in SMD attributable to heterogeneity, and values between 25% and 50% indicate low heterogeneity, whereas between 50% and 75% and >75% indicate moderate and high heterogeneity, respectively. Before the analysis, we formed a prior hypothesis that if heterogeneity existed in the LV function studies, the source could be due to: (1) the difference in the definitions of sepsis (that is, pre-1992 *vs*. post-1992 consensus definition); (2) mortality (for example, in-hospital, 30-day *vs*. 90-day mortality); (3) whether or not the study included patients with severe sepsis; and (4) whether or not patients with history of cardiac disease were excluded. To explore this, we decided that a meta-regression analysis containing the above four covariates should be carried out if the heterogeneity existed [14]. Random permutation test for significance was used to adjust for multiple testings [14].

Small-study effects (also known as 'publication bias') were examined by funnel plots. To avoid subjectivity in the interpretation of funnel plots, we tested for plot symmetry by Egger's regression test [15]. Further assessment of the publication bias was tested using the Duval and Tweedie non-parametric 'trim and fill' method. This method estimates the number and outcomes of missing studies, and adjusts the meta-analysis to incorporate the imputed missing data [16]. Due to the small number of studies, trim and fill analysis was not performed for RV function and dimension analysis.

## Results

### Study identification and selection

**Figure 1:**
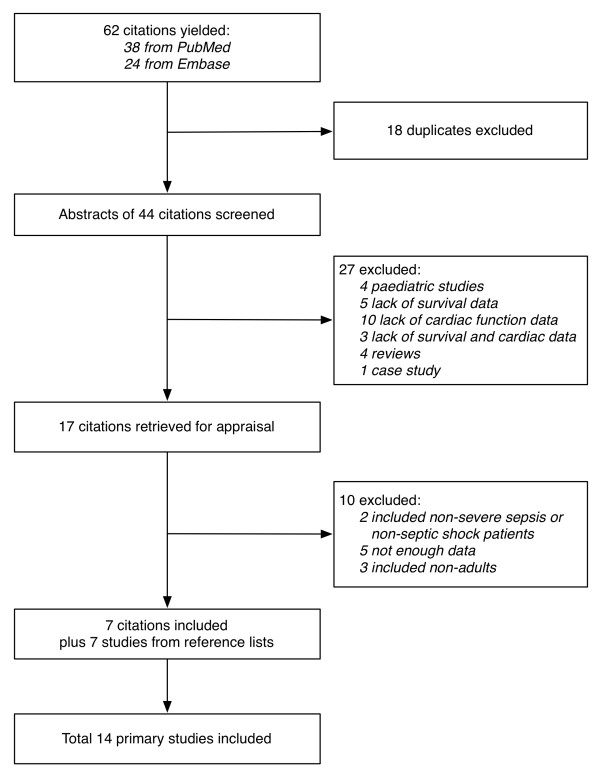
**Workflow of studies identification**.

The literature search yielded 62 citations: 38 from PubMed and 24 from Embase. Eighteen of these were duplicates that appeared in both databases. A total of 44 abstracts were screened. Twenty-seven were excluded mainly due to lack of survival and/or cardiac function data (18 citations) (Figure 1). Seventeen citations were retrieved for appraisal, and a further 10 were excluded due to inclusion of non-adults or non-septic patients in the population or due to insufficient data. Examination of the reference lists of the retrieved studies identified seven relevant citations. Finally, a total of 14 primary studies were included in this systematic review (Table 2) [6-9,17-26].

**Table 2:** Characteristics of included studies.

Author	Year	Country	Study population	**Definition of sepsis and septic shock**^**a**^	Excluded underlying cardiac-related disease	Mean age (years)	Sample size (M/F)
Kimchi et al.	1984	USA	Septic shock	Author	No	65	25 (11/14)
Dhainaut et al.	1988	France	Septic shock	Author	-	47	23 (-/-)
Vincent et al.	1989	Belgium	Septic shock	Author	Yes	-	34 (11/23)
Vincent et al.	1992	Belgium	Septic shock	Author	-	59	68 (45/23)
Jardin et al.	1999	France	Septic shock	Consensus	Yes	55	90 (52/38)
McLean et al.	2007	Australia	Severe sepsis, septic shock	Consensus	No	63	40 (23/17)
Cariou et al.	2008	France	Septic shock	Consensus	Yes	59	10 (7/3)
Etchecopar-Chevreuil et al.	2008	France	Septic shock	Consensus	Yes	56	35 (19/16)
Vieillard-Baron et al.	2008	France	Septic shock	Consensus	Yes	65	67 (50/17)
Sturgess et al.	2010	Australia	Septic shock	Consensus	No	65	21 (13/8)
Furian et al.	2012	Brasil	Severe sepsis, septic shock	Consensus	Yes	51	45 (16/29)
Landesberg et al.	2012	Israel	Severe sepsis, septic shock	Consensus	Yes	61	262 (159/103)
Pulido et al.	2012	USA	Severe sepsis, septic shock	Consensus	Yes	65	106 (53/53)
Weng et al.	2012	China	Septic shock	Consensus	Yes	66	61 (33/28)

^a^Author: Definition of sepsis and septic shock was defined by author but included at least positive culture or documented sources of infection, hypotension, and oligouria. Consensus: The ACCP/SCCM consensus conference definition was used.

## Qualitative summary

### Characteristics of the included studies

The characteristics of the included studies are summarized in Table 2. All studies were published between 1984 and July 2012, and included septic shock patients. Four studies also included patients with severe sepsis [8,9,24,25]. Four studies published prior to 1993 defined septic shock based on clinical diagnosis and included all of the following three criteria: (1) evidence of infection (either sources identified or positive cultures); (2) hypotension; and (3) oliguria [6,17-19]. However, only one of the four studies also included two of the systemic inflammatory response syndrome (SIRS) criteria [19]. Post-1993 studies used the ACCP/SCCM Consensus Conference definition of severe sepsis or septic shock in their recruitment. All eligible patients were recruited in all studies. All studies recruited patients within 24 h of onset of symptoms except for one, which recruited patients 'as early as possible after diagnosis' [25].

Five studies did not list out the sources of sepsis in the cohorts [6-8,17,18]. The sources of sepsis found in the rest of the studies were typical of that normally found in ICU - pneumonia, urosepsis, bacteremia, and abdominal sepsis (Table 3). The exclusion criteria, however, varied greatly among the studies (Table 3). While some studies had clear exclusion criteria, some were unclear or not mentioned [6,20,21]. Most studies excluded patients with cardiac-related disease, three studies did not [9,17,23], and two were silent in this regard [6,18]. One study found their cohort was free from regional wall motion abnormality post recruitment [21]. The types of cardiac-related disease were diverse, but commonly included heart failure, previous myocardial infarction, and/or valvular disease. Four studies excluded patients with suboptimal echo images [9,21,25,26].

**Table 3:** Summary of types of sepsis and exclusion criteria.

Author	Year	Types of sepsis	Exclusion criteria
Kimchi et al.	1984	Not clear, but included ARDS	Aged <18 or >85 years; pregnancy
Dhainaut et al.	1988	-	ARDS
Vincent et al.	1989	Pulmonary and pleura; gastrointestinal; other	HF; MI; recent CPR or cardiac surgery
Vincent et al.	1992	-	-
Jardin et al.	1999	-	Cardiopulmonary disease
McLean et al.	2007	Pulmonary; abdominal; urosepsis; bacteremia; skeletal; skin	Aged <18 years; pregnancy
Cariou et al.	2008	Pneumonia; urosepsis; bacteremia; peritoneal	Suboptimal echo images; none has regional wall motion abnormality
Etchecopar-Chevreuil et al.	2008	Pneumonia; abdominal; urosepsis; skin; other	Other cause of shock; cardiac disease; arrhythmia; moribund status or withhold treatment; leukopenia
Vieillard-Baron et al.	2008	Not clear, but patients had either acute lung injury or ARDS	HF; moribund; did not survive for >48 h
Sturgess et al.	2010	Pulmonary; abdominal; neurologic; fasciitis; catheter-related; mediastinitis	Aged <18 years; valvular disease
Furian et al.	2012	Pulmonary; abdominal; urosepsis	Aged >80 years; HF; liver failure; bone marrow failure; in immunosuppressed state
Landesberg et al.	2012	Pulmonary; gastrointestinal; wound; vascular surgery or limb ischemia; genitourinary; orthopedic; skeletal	Valvular disease; MI
Pulido et al.	2012	-	Aged <18 years; congenital heart disease; valvular disease; coronary heart disease; known abnormality in recent echo
Weng et al.	2012	Pneumonia; bacteremia; peritonitis; other	Aged <18 years; valvular disease; post-thoracic operation; MI; suboptimal echo; moribund

ARDS, acute respiratory distress syndrome; CPR, cardiopulmonary resuscitation; HF, heart failure; MI, myocardial infarction.

The measurements and outcomes are summarized in Table 4. Outcomes were commonly reported as in-hospital mortality. Two studies reported 28- or 30-day all-cause mortality [7,8], and one 90-day all cause mortality [26]. In four studies, the non-survivors died of refractory shock or protracted sepsis [6,18,19,24]. LV functions were commonly reported as LVEF except for one which used LV fractional area contraction. One study used RV stroke volume change as surrogate for RV function whereas the rest used RVEF. Ventricular end-diastolic diameter, area, and volume were variably used for indicating the LV or RV dimension. Some studies indexed the dimension to body surface area (BSA) or height (for example, LV end-diastolic volume index and RV end-diastolic diameter index). The assessment methods used in these studies varied and reflected the standard of assessments at the time - studies published before and up to 1992 used pulmonary artery catheter or radionuclide, and all studies published in 1999 and after utilized echocardiography (either transesophageal or transthoracic) as the standard methods of assessments.

**Table 4:** Measurements.

Author	Year	Outcome (mortality) measure	Cardiac assessment method	LV function (mean ± SD)	LV dimension (mean ± SD)	RV function (mean ± SD)	RV dimension (mean ± SD)
Kimchi et al.	1984	In-hospital all cause	Radionuclide	LVEF (46 ± 16%)	-	RVEF (37 ± 2%)	RVEDVI (98 ± 10 mL/m^2^)
Dhainaut et al.	1988	In-hospital refractory shock	PAC-TD	-	-	RVEF (30 ± 12%)	-
Vincent et al.	1989	In-hospital protracted sepsis	PAC-TD	-	-	RVEF (25 ± 8%)	-
Vincent et al.	1992	In-hospital refractory shock	PAC-TD	-	-	RVEF (38 ± 16%)	RVEDVI (90 ± 31 mL/m^2^)
Jardin et al.	1999	In hospital all cause	TEE	LVEF (49 ± 15%)	LVEDVI^a^ (69 ± 24 mL/m^2^)	-	-
McLean et al.	2007	In hospital all cause	TTE	LVEF (48 ± 15%)	LVEDD (47 ± 10)	-	-
Cariou et al	2008	In hospital all cause	TEE	LVFAC (46 ± 19%)	LVEDA (16 ± 6 cm^2^)	-	-
Etchecopar-Chevreuil et al.	2008	In-hospital all cause	TEE	LVEF (47 ± 20%)	LVEDV (97 ± 25 mL)	-	-
Vieillard-Baron et al.	2008	28-day all cause	TEE	LVEF (51 ± 17%)	LVEDVI^a^ (63 ± 23 mL/m^2^)	-	-
Sturgess et al.	2010	In-hospital all cause	TTE	LVEF (43 ± 14%)	LVEDVI^a^ (67 ± 24 mL/m^2^)	-	-
Furian et al.	2012	In-hospital sepsis	TTE	LVEF (57 ± 13%)	LVEDD/ht (28 ± 3 mm/m)	-	RVD (TTE) (24 ± 4 mm)
Landesberg et al.	2012	In-hospital all cause	TTE	LVEF (59 ± 11%)	LVEDD (46 ± 10 mm) LVEDVI^a^ (56 ± 18 mL/m^2^)	RVSV change (7.5 ± 3.7 mL)	RVEDA (21 ± 6 cm^2^)
Pulido et al.	2012	30-day all cause	TTE	LVEF (57 ± 15%)	LVEDD (46 ± 10 mm)	-	-
Weng et al.	2012	90-day all cause	TTE	LVEF (55 ± 16%)	LVEDV (72 ± 24 mL)	-	-

^a^I represents index to body surface area.

LVEDA, LV end-diastolic area; LVEDD, LV end-diastolic diameter; LVEDV, LV end-diastolic volume; LVEF, LV ejection fraction; LVFAC, LV fractional area contraction; PAC-TD, Pulmonary artery catheter-thermodilution; Radionulcide, radionuclide-gated cardiac cineangiography; RVD, RV diameter; RVEDA, RV end-diastolic area; RVEDVI, RV end-diastolic volume index; RVEF, RV ejection fraction; RVSV change, RV stroke volume change; TEE, transesophageal echocardiography; TTE, transthoracic echocardiography;.

## Quantitative summary

### Left ventricle (function and dimension) and mortality

**Figure 2:**
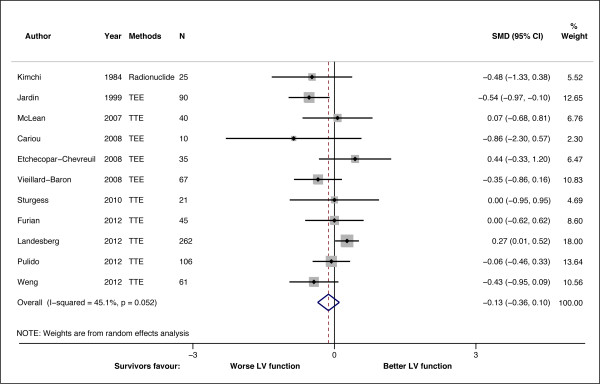
**Standardized mean difference (SMD) for LV function in survivor and non-survivors**. Forest plot showing the SMDs for different studies and the overall SMD. Negative or positive SMDs imply smaller or larger LVEF or LVFAC in the survivors. TEE, transesophageal echocardiography; TTE, transthoracic echocardiography.

**Figure 3:**
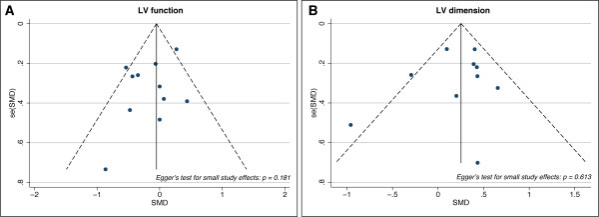
**Funnel plot for small-study effects**. Funnel plot showing possible publication bias due to small-study effects. **(A) **Funnel plot for LV function studies. **(B) **Funnel plot for LV dimension study.

Figure 2 shows the Forest plot of the pooled results of the weighted difference of the 11 studies. Total sample size was 762. The pooled SMD was -0.13 (-0.36 to 0.10), indicating there was no significant difference between the survivor and non-survivors in terms of LV function (*P *= 0.284). Statistical heterogeneity was mild (I^2 ^= 45.1%; *P *= 0.052). Meta-regression analysis shows that none of the covariates proposed in our prior hypothesis contributed to the heterogeneity observed (*P *= 0.201) (Table 5). The symmetry of funnel plot suggested the absence of small study effects or publication bias (Egger's test for small-study effects: *P *= 0.181) (Figure 3A). Trim and fill analysis also did not impute any missing studies.

**Table 5:** Meta-regression analysis for potential covariates contributing to heterogeneity.

Covariates	Coefficient	95% CI	*P *value
*Outcome measures* In-hospital *vs*. 30-day/90-day mortality	-0.19	-0.66 to 0.29	0.372
*Patient types* Septic shock *vs*. severe sepsis and septic shock	0.42	-0.03 to 0.87	0.062
*Definition* Consensus *vs*. non-consensus	-0.26	-1.62 to 1.10	0.66
*Exclusion of existing heart-related condition*	0.027	-0.78 to 0.84	0.94

**Figure 4:**
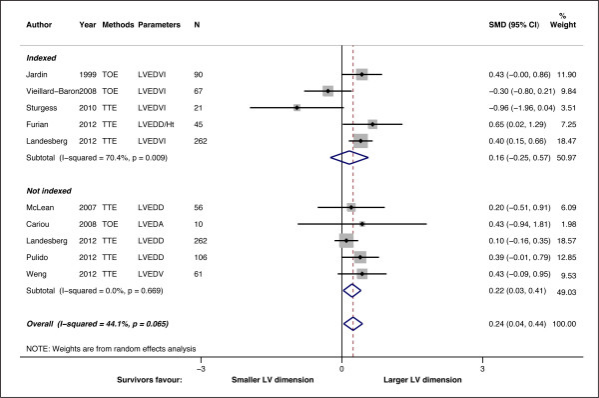
**Standardized mean difference (SMD) for LV dimension in survivor and non-survivors**. Forest plot showing the SMDs for different studies and the overall SMD. Studies were divided into two groups: Indexed, where LV dimension were indexed to body surface area or height, and not indexed. Subtotal SMD for each group are also shown. Negative or positive SMDs imply smaller or larger LV dimension in the survivors.

The pooled SMD for LV dimensions indexed to BSA or body height was 0.16 (-0.25 - 0.57), indicating that there was no significant difference in LV dimension between survivors and non-survivors (*P *= 0.439) (Figure 4). However, the non-indexed dimensions were mildly increased in survivors (SMD = 0.22 (0.03 - 0.41); *P *= 0.023). When pooled together (718 patients), the overall SMD for LV dimension demonstrated a mild but significant increase (SMD = 0.24 (0.04 - 0.44); *P *= 0.019), indicating survivors had larger LV dimensions. Overall heterogeneity was mild (I^2 ^= 44.1%, *P *= 0.065), but moderate heterogeneity was present among those studies utilizing indexed dimension (I^2 ^= 70.4%, *P *= 0.009). No small-study effects or publication bias were found (*P *= 0.613) (Figure 3B). Trim and fill analysis imputed one 'missing' study, but did not alter the overall SMD (0.21 (0.14 - 0.41)) and heterogeneity (I^2 ^= 45.3%, *P *= 0.05) significantly.

### Right ventricle (function and dimension) and mortality

**Figure 5:**
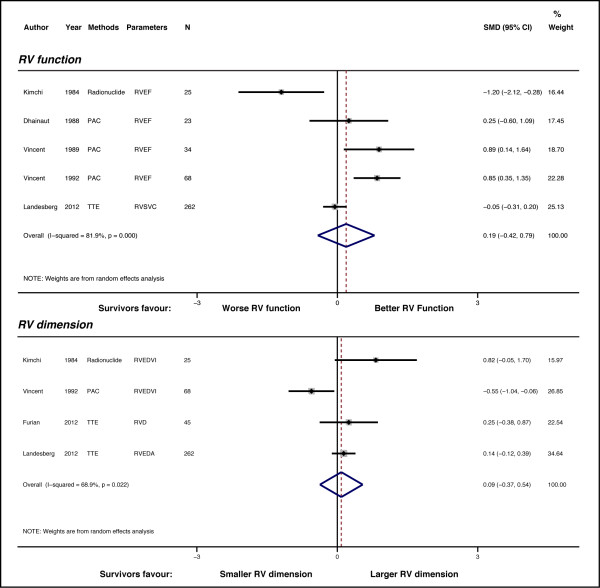
**Standardized mean difference (SMD) for RV function and dimension in survivor and non-survivors**. Forest plot showing the SMDs for different studies and the overall SMD. Upper panel, Forest plot for RV function; lower panel, Forest plot for RV dimension. Negative or positive SMDs imply smaller or larger LV dimensions in the survivors.

Only five studies (a total of 412 patients) were included for RV function analysis. There was no significant difference in RV function between survivors and non-survivors (pooled SMD = 0.19 (-0.42 - 0.79); *P *= 0.545). The heterogeneity was high (I^2 ^= 81.9%, *P *< 0.001) (Figure 5, upper panel). We noticed that one of the study could be an outlier (17), however, excluding the study did not improve the heterogeneity probably due to the small number of studies (I^2 ^= 77.9%; *P *= 0.004). There was also no significant difference in RV dimension between the survivors and non-survivors (pooled SMD = 0.09 (- 0.37 - 0.54); *P *= 0.713) (Figure 5, lower panel). Heterogeneity was also high (I^2 ^= 68.9%, *P *= 0.022). Test for small-study effects was not performed due to the small number of studies.

## Discussion

This meta-analysis provides pooled estimates of RV and LV function and dimension in patients with severe sepsis and septic shock from 14 individual studies. Using a set of well-defined inclusion criteria, we found that there was not enough evidence to support the view that survivors from severe sepsis or septic shock suffered from early LV dysfunction. When indexed to BSA or body height, the LV dimensions were similar in survivors and non-survivors. However, un-indexed dimensions and pooled results suggested that the LV were mildly enlarged in survivors. There were no statistical significant differences in RV function or dimensions between survivors and non-survivors.

LV depression is a well-established phenomenon among patients with severe sepsis or septic shock [27]. Multiple mechanisms are believed to be responsible for sepsis-induced LV depression, including mitochondrial dysfunction, oxidative stress, inflammatory actions, and myocardial injury [28]. Using radionuclide cineangiography, Parker and colleagues observed that survivors from septic shock developed acute LV and RV depression and dilatation which normalized with recovery, hence the term 'reversible myocardial depression' [4,5]. Similar findings were made by Jardin [21]. Yet, a number of subsequent studies failed to reproduce Parker's results. For example, we analyzed the 1984 study results from Kimchi et al. and could not find any significant differences in LVEF or RVEF between survivors and non-survivors from septic shock (43 ± 13% *vs*. 51 ± 22% for LVEF (*P *= 0.232) and 35 ± 2% *vs*. 39 ± 5% for RVEF (*P *= 0.439)) [17]. Vincent et al., on the other hand, showed that survivors from septic shock had better right ventricle function [6,19]. There are several explanations for the discordances reported by these studies, including sample size, definition of sepsis and septic shock, study population, types of cardiac investigations, and effects of therapy.

### Sample size

Earlier studies usually had smaller sample sizes and significant group size imbalance. For example, the original study by Parker et al. had only 20 patients with seven non-survivors, Kimchi et al. had 25 with only eight non-survivors, and Vincent et al. had 34 patients (23 non-survivors) with septic shock. The main problems arise from small studies are that they tend to result in imprecise estimates and are more likely to find extreme results. The latter of these can account for inter-study discordant. To detect a 10% difference in LVEF between two groups (assuming a SD of 15%), one needs at least 70 patients (35 in each group) to achieve 80% power. A 5% difference will need over 300 patients in total. While more recent studies tended to be larger in size, few of them had the necessary sample size to provide a conclusive result.

### Definition of severe and time of recruitment

Some primary studies used different definitions of septic shock for recruiting patients, and those published before the Consensus definitions defined septic shock differently. Even with the publication of the Consensus definitions, the process has not been made easier [10]. For instance, depending on whether a restricted or a liberal approach is used, applying the consensus definition resulted in a range of incidences of severe sepsis (6% to 27%) and septic shock (4% to 9%) in the same study [29]. This highlighted the difficulty in recruiting patients in sepsis studies. Not only could the differences in definitions and recruitment stringency affect the type of patients recruited, but the timing of recruitment could also contribute. One typical situation is that not all SIRS criteria are present at start of infection, which itself is difficult to define [30]. Although some studies described the times of recruitment and when the first assessments were done, others were not so explicit probably due to the difficulty in defining the onset of sepsis, which might have occurred before admission to ICU. Arguably, some studies might report the worst values, while other might have reported the values on Day 1. Recruiting patients of different types or at different stages could inevitably affect the composition of study population, hence the generalizability.

### Study population

To have a homogeneous study population across different studies is a challenge in systematic reviews. Heterogeneity of study populations can arise from two sources: (1) inclusion; and (2) exclusion. Although the study populations in all of the studies were severe sepsis and septic shock, the sources of sepsis varied. Fortunately, the sources of sepsis found were comparable in most of the included studies, and were typical to that found in general ICU. On the other hand, the exclusion criteria varied widely among the studies. The most common exclusion factor was the presence of pre-existing cardiac conditions. Although it is understandable that excluding these patients would reduce the risk of type I error (false-positives) of finding sepsis-induced myocardial depression, such exclusion could be a curb on giving the answer if pre-existing cardiac conditions would pre-dispose patients to sepsis-induced myocardial depression and/or increase the risk of death. In the original study by Parker et al., four of the 20 patients had underlying cardiac conditions (coronary heart disease and cardiomyopathy), but this did not increased the risk of death (OR = 0.6; 95% CI = 0.06 to 5.6) [4]. In our previous study, none of the 12 patients with pre-existing cardiac conditions developed sepsis-induced myocardial depression and the presence of cardiac conditions did not increase the risk of death statistically (OR = 2.4; 95% CI = 0.5 to 10.7) [9]. Similar findings were found in a study by ver Elst et al. where previous myocardial infarct did not increase mortality in septic shock (OR = 2.1; 95% CI = 0.5 to 8.3) [31]. These studies suggested that pre-existing cardiac conditions neither altered the risk of sepsis-induced myocardial depression nor increased the risk of death in severe sepsis and septic shock. As the total sample size was still small in these studies, more research is required in this area.

### Assessment methods

In the last three decades, there has been a clear shift in assessment methods for EFs in these studies - from invasive and to non-invasive. Temporally, radionuclide angiography was the mainstream in the earliest research, followed by pulmonary artery catheter thermodilution, and finally by echocardiography in modern era. Some doubts existed in relation to the comparability of LVEF obtained by echocardiography and radionuclide angiography. Takenaka et al. found that LVEF measurements by the two methods could yield different estimates as a result of the state of the patients such as anxiety [32]. More recent studies, however, demonstrated that the two methods have good comparability [33,34]. The comparability of RVEF by thermodilution and radionuclide however was not as good [35]. Of note, Vincent et al. found that RVEF from thermodilution could be underestimated in the presence of tricuspid regurgitations [36]. The complicated shape of the right ventricle precludes the use of echocardiography to measure RVEF. Due to a lack of strong evidence to support comparability among the three methods, readers quoting EFs for survivors and non-survivors need to mention the methods used.

### Effects of therapy

Depending on the time of cardiac assessments, fluid resuscitation had the potential effects of altering the ventricular dimensions and possibly function. However, most of the studies were not clear whether fluid had been administered before or after measurements. The use of vasopressors and/or inotropes is another factor that could affect the findings. Vieillard-Baron et al. found that while some (39%) patients with septic shock exhibited 'primary' global hypokinesia (depressed LV function) at admission, some (21%) developed 'secondary' global hypokinesia 24 to 48 h after hemodynamic support by norepinephrine [7]. This 'secondary' hypokinesia could be partially reversed by dobutamine. The authors could not rule out a causative relationship between noradrenaline and hypokinesia, but pointed out that the hypokinesia could be the direct deleterious effects of the associated increase in afterload on a latent dysfunctioning heart in sepsis. If this is true, then studies that had their initial assessments done within 24 h of onset of symptoms could have underestimated the extent of myocardial depression in their cohorts. This could have implications in the interpretation of myocardial depression and mortality.

Mechanical ventilation is another potential confounding factors that could affect the ventricular function and dimension in sepsis via heart-lung interactions. The change in ventilation practice since the ARDSNet study in 2000 [37] is a possible bias or confounders for ventricular function and dimension. However, the effects of mechanical ventilation, in particular protective ventilation, were not explored due to a lack of consistent ventilation data. In this regards, it is noteworthy that most eligible studies contained a mixed population of sepsis, instead of a pure population with acute lung injury or acute respiratory distress syndrome. Hence, the ventilator settings might be different for different patients.

### Effects of indexation on LV dimensions

Our meta-analysis found that when LV dimensions were indexed against BSA or body height, no differences between survivors and non-survivors were found in the pooled results. However, the survivors displayed mildly dilated LV ventricles when non-indexed dimensions were used. Of interest, in Landesberg et al.'s study, non-indexed LVEDD resulted in non-significant results between the survivors and non-survivors, whereas indexed LVEDV led to significant different results. Differences in using indexed and non-indexed parameters are to be expected. While it is natural for heart size to follow both body size, body composition and physiological demands (for example, exercise) to accommodate the greater metabolic needs, the heart is also capable of adapting itself to acute conditions, for example, dilating acutely with expanded intravascular volume. According to our findings, the non-indexed dimensions were more consistent across studies when compared to indexed dimensions. Depending on which parameter is used, this clearly has an impact on study interpretations. More studies are needed in this area.

### Excluded studies

Most studies were excluded due to a lack of sufficient data. One relevant study was excluded because it reported the results as median (25th/75th percentile), and the author did not provide the data in mean (± SD) when requested [38]. Nevertheless, the authors in that study found no difference in LVEF between the survivor and non-survivor groups. Unfortunately, we have to exclude all studies published by Parker and colleagues due to the inclusion of non-adults in their cohorts [4,39,40]. It was also unclear if the patients were recruited consecutively or if all the patients within the study period were recruited. Another interesting point to note about Parker's 1984 studies is that the cohort consisted of a large proportion (14 out of 20) of patients with underlying malignancy, whether or not the cardiac function or dimensions of these patients were already affected by the chemotherapy was unknown.

We also excluded studies that used other non-global estimates for LV or RV function, such as tissue Doppler as it only measures one segment of the myocardium [30].

### Suggestions for future studies

Considering the challenges above, we suggest future studies should be prospective in nature and use the Consensus definition for recruitment. To improve generalizability, exclusion criteria should not be too restrictive and inclusion of patients with underlying cardiac conditions should be considered. To handle possible confounding effects of underlying cardiac conditions, *a priori *plan of subgroups analyses can be made. The sample size should be estimated beforehand based on published data. To facilitate comparisons between studies, results should report the mean (SD), plus median (95% CI) if necessary. Echocardiography remains as the preferred methods for assessment at present. Although echocardiography cannot assess RVEF, other estimates of RV function (for example, tissue Doppler) can be used instead. Treatments (vasopressors and inotropes) given, and whether or not the patient received fluid, at the time of assessment should be documented. Serial measurements, at least for the first 2 to 3 days and after fluid optimization and norepinephrine administration, should be performed. While time-to-event survival analysis is the preferred method of analysis, ICU and in-hospital mortality should be reported at the very least.

### Limitations of the meta-analysis

The main purpose of meta-analysis is to summarize the results from smaller studies in a systematic and unbiased manner. Hence, the quality and conclusion of meta-analysis depends on the quality of included studies. The variability in study designs is one of the most concerned factor in meta-analysis. In this study we addressed a number of factors that could possibly explained results heterogeneity and might also affect the quality of this meta-analysis: sample size, definition of sepsis and septic shock, study population, types of cardiac investigations, and effects of therapy. To minimize results heterogeneity, we used a list of objective and unbiased recruitment criteria to identify appropriate studies. However, since meta-analysis is retrospective in nature, statistical manipulation could neither save the differences in study designed nor improve the studies' quality. As such, we are unable to address several important issues due to inconsistent study designs and lack of data: pre-admission ventricular function, the precise effects of early administration of vasopressors and inotropes, the relationship between load-independent myocardial function index and mortality. A definitive answer to these questions will require a large and well-designed prospective study.

## Conclusions

By pooling 14 studies together, we managed to achieve a reasonable sample size (>750) to examine the question whether or not early ventricular dysfunction or dilatation is associated with better mortality. We could not find convincing evidence that initial low LVEF is associated with better mortality in patients with severe sepsis or septic shock. Although survivors seemed to have larger left ventricular size, the indexed LV dimensions were similar in both groups. RVEF and RV dimension, on the other hand, are not associated with mortality. In view of these results, perhaps we should avoid using ventricular dysfunction, especially LVEF, for prognosticating patients with severe sepsis or septic shock. Since many septic patients displayed reversible myocardial depression, LVEF in these patients could be a mere reflection of the balance between ventricular function and loading conditions [41].

## Key messages

• Most of the eligible primary studies were observational descriptive studies with limited sample size. None of the studies were designed as prognosis studies using ejection fractions or ventricular dimensions as prognosticator.

• Pooled results do not suggest survivors from severe sepsis or septic shock had lower ejection fractions.

• Overall results seemed to suggest survivors exhibited slightly larger LV dimensions but pooled indexed LV dimensions were similar in survivors and non-survivors.

• A more definitive larger trial is needed to confirm these findings and to establish if indexed or non-indexed dimensions should be used in clinical context.

## Abbreviations

BSA: body surface area; EF: ejection fraction; ICU: intensive care unit; LV: left ventricle; LVEDD: left ventricular end-diastolic diameter; LVEDV: left ventricular end-diastolic volume; RV: right ventricle; RVEDA: right ventricular end-diastolic area; RVEDD: right ventricular end-diastolic diameter; RVEDV: right ventricular end-diastolic volume; SIRS: systemic inflammatory response syndrome; SMD: standardized mean difference.

## Conflicting interests

The authors declare that they have no competing interests.

## Authors' contributions

SJH and ASM initiated and designed the study. SJH and MN performed the literature search, screened and appraised the papers. SJH performed the statistical analysis. SJH, MN, and ASM drafted the manuscript. All authors have read and approved the manuscript for publication.

## References

[B1] McLean AS Down but not out: myocardial depression in sepsis Crit Care 2012 17 132 10.1186/cc11367 22748042 PMC3580648

[B2] Weisel RD Vito L Dennis RC Valeri CR Hechtman HB Myocardial depression during sepsis Am J Surg 1977 17 512 521 10.1016/0002-9610(77)90141-6 848686

[B3] Hoffman MJ Greenfield LJ Sugerman HJ Tatum JL Unsuspected right ventricular dysfunction in shock and sepsis Ann Surg 1983 17 307 319 10.1097/00000658-198309000-00007 6615053 PMC1353298

[B4] Parker MM Shelhamer JH Bacharach SL Green MV Natanson C Frederick TM Damske BA Parrillo JE Profound but reversible myocardial depression in patients with septic shock Ann Intern Med 1984 17 483 490 10.7326/0003-4819-100-4-483 6703540

[B5] Parker MM McCarthy KE Ognibene FP Parrillo JE Right ventricular dysfunction and dilatation, similar to left ventricular changes, characterize the cardiac depression of septic shock in humans Chest 1990 17 126 131 10.1378/chest.97.1.126 2295231

[B6] Vincent JL Gris P Coffernils M Leon M Pinsky M Reuse C Kahn RJ Myocardial depression characterizes the fatal course of septic shock Surgery 1992 17 660 667 1595062

[B7] Vieillard-Baron A Caille V Charron C Belliard G Page B Jardin F Actual incidence of global left ventricular hypokinesia in adult septic shock Crit Care Med 2008 17 1701 1706 10.1097/CCM.0b013e318174db05 18496368

[B8] Pulido JN Afessa B Masaki M Yuasa T Gillespie S Herasevich V Brown DR Oh JK Clinical spectrum, frequency, and significance of myocardial dysfunction in severe sepsis and septic shock Mayo Clin Proc 2012 17 620 628 10.1016/j.mayocp.2012.01.018 22683055 PMC3538477

[B9] McLean AS Huang SJ Hyams S Poh G Nalos M Pandit R Balik M Tang B Seppelt I Prognostic values of B-type natriuretic peptide in severe sepsis and septic shock Crit Care Med 2007 17 1019 1026 10.1097/01.CCM.0000259469.24364.31 17334249

[B10] American College of Chest Physicians/Society of Critical Care Medicine Consensus Conference: definitions for sepsis and organ failure and guidelines for the use of innovative therapies in sepsis Crit Care Med 1992 17 864 874 10.1097/00003246-199206000-00025 1597042

[B11] Centre for Evidenced Based Medicine http://www.cemb.net (accessed on 3 August, 2012)

[B12] DerSimonian R Laird N Meta-analysis in clinical trials Controlled Clinical Trials 1986 17 177 188 10.1016/0197-2456(86)90046-2 3802833

[B13] Higgins JPT Thompson SG Deeks JJ Altman DG Measuring inconsistency in meta-analysis BMJ 2003 17 557 560 10.1136/bmj.327.7414.557 12958120 PMC192859

[B14] Higgins JPT Thompson SG Controlling the risk of spurious findings from meta-regression Stat Med 2004 17 1663 1682 10.1002/sim.1752 15160401

[B15] Egger M Davey Smith G Schneider M Minder C Bias in meta-analysis detected by a simple, graphical test BMJ 1997 17 629 634 10.1136/bmj.315.7109.629 9310563 PMC2127453

[B16] Duval S Tweedie R A nonparametric "trim and fill" method of accounrting for publication bias in metaanalysis J Am Stat Assoc 2000 17 89 98

[B17] Kimchi A Ellrodt AG Berman DS Riedinger MS Swan HJC Murata GH Right ventricular performance in septic shock: A combined Radionuclide hemodynamic study J Am Coll Cardiol 1984 17 945 951 10.1016/S0735-1097(84)80055-8 6491086

[B18] Dhainaut JF Lanore JJ de Gournay JM Huyghebaert MF Brunet F Villemant D Monsallier JF Right ventricular dysfunction in patients with septic shock Intensive Care Med 1988 17 488 491 3403793 10.1007/BF00256967

[B19] Vincent J-L Frank CRN Contempre B Kahn RJ Right ventricular dysfunction in septic shock: assessment by measurements of right ventricular ejection fraction using the thermodilution technique Acta Anaesthesiol Scand 1989 17 34 38 2916389 10.1111/j.1399-6576.1989.tb02856.x

[B20] Jardin F Fourme T Page B Loubières Y Vieillard-Baron A Beauchet A Bourdarias JP Persistent preload defect in severe sepsis despite fluid loading. A longitudinal echocardiographic study in patients with septic shock Chest 1999 17 1354 1359 10.1378/chest.116.5.1354 10559099

[B21] Cariou A Pinsky MR Monchi M Laurent I Vinsonneau C Chiche JD Charpentier J Dhainaut JF Is myocardial adrenergic responsiveness depressed in human septic shock? Intensive Care Med 2008 17 917 922 10.1007/s00134-008-1022-y 18259725

[B22] Etchecopar-Chevreuil C Francois B Clavel M Pichon N Gastinne H Vignon P Cardiac morphological and functional changes during early septic shock: a transesophageal echocardiographic study Intensive Care Med 2008 17 250 256 10.1007/s00134-007-0929-z 18004543

[B23] Sturgess DJ Marwick TH Joyce C Jenkins C Jones M Masci P Stewart D Venkatesh B Prediction of hospital outcome in septic shock: a prospective comparison of tissue Doppler and cardiac biomarkers Crit Care 2010 17 R44 10.1186/cc8931 20331902 PMC2887156

[B24] Furian T Aguiar C Prado K Ribeiro RV Becker L Martinelli N Clausell N Rohde LE Biolo A Ventricular dysfunction and dilation in severe sepsis and septic shock: relation to endothelial function and mortality J Crit Care 2012 17 319.e9 319.e15 10.1016/j.jcrc.2011.06.017 21855287

[B25] Landesberg G Gilon D Meroz Y Georgieva M Levin PD Goodman S Avidan A Beeri R Weissman C Jaffe AS Sprung CL Diastolic dysfunction and mortality in severe sepsis and septic shock Eur Heart J 2012 17 895 903 10.1093/eurheartj/ehr351 21911341 PMC3345552

[B26] Weng L Liu YT Du B Zhou JF Guo XX Peng JM Hu XY Zhang SY Fang Q Zhu WL The prognostic value of left ventricular systolic function measured by tissue Doppler imaging in septic shock Crit Care 2012 17 R71 10.1186/cc11328 22554063 PMC3580613

[B27] Hochstadt A Meroz Y Landesberg G Myocardial dysfunction in severe sepsis and septic shock: more questions than answers? J Cardiothorac Vasc Anesth 2011 17 526 535 10.1053/j.jvca.2010.11.026 21296000

[B28] Balija TM Lowry SF Lipopolysaccharide and sepsis-associated myocardial dysfunction Curr Opin Infect Dis 2011 17 248 253 10.1097/QCO.0b013e32834536ce 21378563

[B29] Klein Klouwenberg PM Ong DS Bonten MJ Cremer OL Classification of sepsis, severe sepsis and septic shock: the impact of minor variations in data capture and definition of SIRS criteria Intensive Care Med 2012 17 811 819 10.1007/s00134-012-2549-5 22476449

[B30] Vincent JL Dear SIRS, I'm sorry to say that I don't like you Crit Care Med 1997 17 372 374 10.1097/00003246-199702000-00029 9034279

[B31] ver Elst KM Spapen HD Nguyen DN Garbar C Huyghens LP Gorus FK Cardiac troponins I and T are biological markers of left ventricular dysfunction in septic shock Clin Chem 2000 17 650 657 10794747

[B32] Takenaka A Iwase M Sobue T Yokota M The discrepancy between echocardiography, cineventriculography and thermodilution. Evaluation of left ventricular volume and ejection fraction Int J Card Imaging 1995 17 255 262 10.1007/BF01145194 8596064

[B33] Galasko GI Basu S Lahiri A Senior R Is echocardiography a valid tool to screen for left ventricular systolic dysfunction in chronic survivors of acute myocardial infarction? A comparison with radionuclide ventriculography Heart 2004 17 1422 1426 10.1136/hrt.2003.027425 15547019 PMC1768568

[B34] Almeda FQ Hendel RC Macioch JE Sandelski J Parrillo JE Meyer PM Johnson M Daniels ML Go VU Feinstein SB Comparison of echocardiography using tissue harmonics and contrast harmonics with radionuclide angiography for the assessment of left ventricular function J Investig Med 2003 17 366 372 10.2310/6650.2003.8952 14686640

[B35] Starling RC Binkley PF Haas GJ Hatton PS Wooding-Scott M Thermodilution measures of right ventricular ejection fraction and volumes in heart transplant recipients: a comparison with radionuclide angiography J Heart Lung Transplant 1992 17 1140 1146 1333800

[B36] Vincent JL Thirion M Melot C Leeman M Reuse C Lenaers A Discrepancy between thermodilution and radionuclide right ventricular ejection fraction measurements: the importance of tricuspid regurgitation Acute Care 1986 17 49 51 3434158

[B37] The acute respiratory distress syndrome network Ventilation with lower tidal volumes as compared with traditional tidal volumes for acute lung injury and the acute respiratory distress syndrome N Engl J Med 2000 17 1301 1308 10793162 10.1056/NEJM200005043421801

[B38] Post F Weilemann LS Messow CM Sinning C Munzel T B-type natriuretic peptide as a marker for sepsis-induced myocardial depression in intensive care patients Crit Care Med 2008 17 3030 3037 10.1097/CCM.0b013e31818b9153 18824903

[B39] Parker MM Suffredini AF Natanson C Ognibene FP Shelhamer JH Parrillo JE Responses of left ventricular function in survivors and nonsurvivors of septic shock J Crit Care 1989 17 19 25 10.1016/0883-9441(89)90087-7

[B40] Parker MM Ognibene FP Parrillo JE Peak systolic pressure/end-systolic volume ratio, a load-independent measure of ventricular function is reversibly decreased in human septic shock Crit Care Med 1994 17 1955 1959 7988132

[B41] Vieillard Baron A Schmitt JM Beauchet A Augarde R Prin S Page B Jardin F Early preload adaptation in septic shock? A transesophageal echocardiographic study Anesthesiology 2001 17 400 406 10.1097/00000542-200103000-00007 11374597

